# Status of Iran’s Primary Health Care System in Terms of Health Systems Control Knobs: A Review Article

**Published:** 2017-09

**Authors:** Jafar Sadegh TABRIZI, Faramarz POURASGHAR, Raana GHOLAMZADEH NIKJOO

**Affiliations:** 1. Health Services Management, Tabriz Health Services Management Research Center, Dept. of Health Services Management, Faculty of Management and Medical Informatics, Tabriz University of Medical Sciences, Tabriz, Iran; 2. Road Traffic Injury Research Center and Dept. of Medical Informatics, School of Management and Medical Informatics, Tabriz University of Medical Sciences, Tabriz, Iran; 3. Iranian Center of Excellence in Health Management, Dept. of Health Services Management, School of Management and Medical Informatics, Tabriz University of Medical Sciences, Tabriz, Iran

**Keywords:** Primary health care, Health system, Control knob, Iran

## Abstract

**Background:**

After the establishment of Primary Health Care (PHC) program in Iran, health indicators have improved every year. This progress was so rapid that a number of shortcomings and weaknesses of the PHC program remained silent behind its successes. This study aimed to assess the status of Iran’s PHC system (strengths, weaknesses, opportunities and threats) in terms of health system’s control knobs.

**Methods:**

The search was conducted through two English ‘databases of Web of Knowledge and PubMed, two English publications of Science Direct and Springer and two Persian databases of Magiran and SID. Keywords were selected from MeSH and included primary health care, PHC and Iran in both Persian and English. No time limit was considered.

**Results:**

Iran’s PHC system has numerous successes in dealing with health system’s control knobs; which largely part of that related to the health network implementation, the role of Behvarz, improvement of health indicators in rural areas and the elimination of urban-rural inequality, but there are some weaknesses, opportunities and threats in the Iranian PHC system as well.

**Conclusion:**

By considering socio-economic changes the current structure of PHC system needs to be reformed to coordinate with phenomenon of chronic diseases, accidents and aging. The current information system in PHC does not provide the required information for decision makers and policy makers so it needs to be transformed to the electronic system with unique electronic health file for individuals.

## Introduction

The most important historical event in the development and delivery of health services is the international community’s decision on the adoption of Primary Health Care (PHC) in order to achieve community justice in access to basic health services (1,2). The general goal was health for all by the year 2000 and the key to achieve such goal is defined PHC services (1, 3–5). Around 1974, Iran began studying on health system. The first signs of the establishment of PHC appeared in 1979, but the full deployment of health care networks occurred in 1985 (6–8).

According to the structure of PHC system in Iran, each village (sometimes a collection of villages) has a health-house, staffed by trained health care provider named Behvarz (Multi-purpose health care worker), who covers health care of 1200 inhabitants. These health-houses are the first level of contact between families and the health system. In the big villages in addition to health-houses, there are rural health centers. Their staff is a qualified physician and a team of up to 10 health workers that provide care for more complex health problems. Each rural health center covers almost 7000 inhabitants. In urban areas health posts and health centers provide similar services as health-houses and rural health centers. This network is managed by district health centers, under the supervision of medical sciences universities. In each province, there is at least one Medical Sciences University (8, 9).

After the establishment of PHC program, health indicators continued to improve every year. This progress was so rapid, therefore, number of shortcomings and weaknesses of the PHC program was not apparent, or remained silent behind the successes of it. Despite the rapid development of the system and obtaining magnificent achievements, this system needs to be modified because of the gradually changing the pattern of the disease, community’s needs and also changing the epidemiological structures (8).

The most important factors that determine the outcomes of each health system are control knobs (10). Control knobs are types of ‘tools’ available for all managers and policy makers to determine health system status. Five control knobs are financing, payment, organizing, regulation and behavior (11). Health system policy makers usually focus on the final goals, which are providing healthcare, responsiveness and fairness in financing. Their important instruments to reach these goals are control knobs. Control knobs’ framework first expressed (12) and further refined (11).

Control knobs are used for understanding the health system and its performance (13) and by considering these facts that most previous studies were about success or weaknesses of Iran’s PHC system and is not done yet any comprehensive study on determining system status in terms of strengths, weaknesses, opportunities and threats from the perspective of health system control knobs, the objective of this study was to determine the status of Iran’s PHC system in terms of health systems five control knobs.

## Materials and Methods

The search was carried out for published literature in two English databases of Web of Knowledge and PubMed, two English publications of Science Direct and Springer and two Persian information resources of Magiran and SID, as well as other sources such as books, reports, websites of the Ministry of Health and Medical Education and some international organizations such as WHO and World Bank.

All published evidence about PHC in Iran aimed to review, using the “primary health care” and “PHC” MeSH terms and “Iran” as keywords in both Persian and English databases. No time limit was considered and the search was conducted from 29^th^ of Aug 2014 until 20^th^ of Nov 2014. A repeated search was performed in Aug 2016 to update the data.

**Fig. 1:**
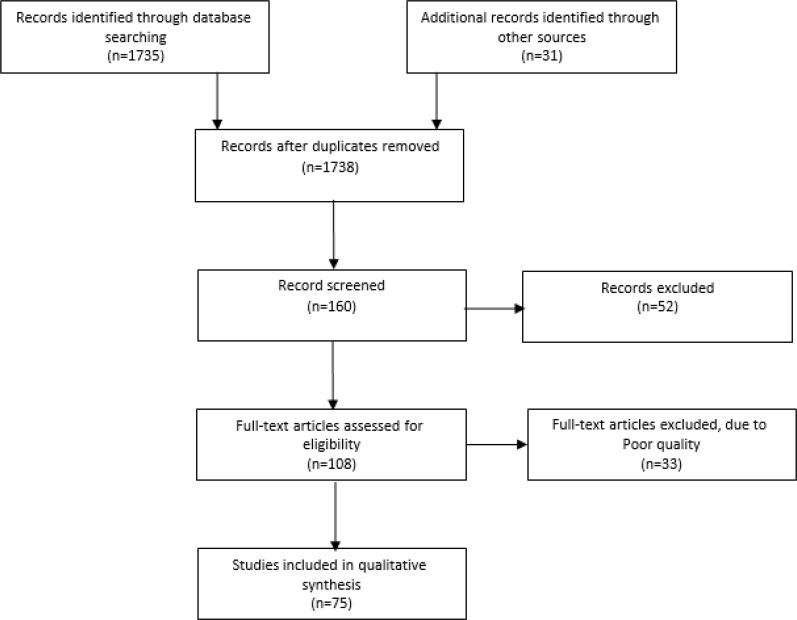
Process of study selection

Cross-sectional studies, qualitative studies, reviews and meta-analyses that reported evidence about Iran’s health system status were assessed. All papers that described strengths, weaknesses, opportunities and threats of Iran’s PHC system were included in the study. Studies that only had described Iran’s PHC structure were excluded. A large number of documents were obtained from the search (Fig. 1).

Two authors independently assessed retrieved relevant evidence for further assessment. We used Critical Appraisal Skills Program (CASP) tools to review qualitative studies and systematic reviews (14), for cross-sectional studies, the “National Heart, Lung and Blood Institute, quality Assessment tool for observational cohort and cross-sectional studies” were used (15).

Any disagreement between reviewers was resolved by consensus (11, 16, 17). After reading the selected evidence, the strengths, weaknesses, opportunities and threats of the Iranian PHC system were identified by two authors using Robert and Partner’s control knobs classification in 2004 (10).

## Results

Overall, 75 studies were fully studied. The strengths, weaknesses, opportunities and threats of the Iranian PHC system based on health system’s control knobs are shown in Table 1.

**Table 1:** Iran’s PHC system’s status based on health system’s control knobs

**Health system status**	**Internal Status**		**External Status**	
**Control Knobs Organizing**	**Strengths**	**Weaknesses**	**Opportunities**	**Threats**
	-Wide PHC network in the country (11, 21, 25,33, 34, 35, 37, 39, 41, 75) -Full access of rural population to PHC (26, 36, 38, 43, 51, 54) -Transferring unenforceable programs from environmental levels to higher levels (40) -Introducing Behvarz (20, 40, 79) -Organizing health volunteers in health posts (9) -Development of Family physician program and referral systems in villages (23, 29, 31, 41, 43) -Integrating the management of non-communicable diseases in the villages (9, 52, 69, 80)	-Mechanical organizational structure at local level (50) -Lack of change in PHC structure along with changes in health needs (8, 17, 18, 30, 32, 45–49) -Formal structure of the system (17) -inadequate development of PHC in urban areas (17,25,29,30,32 43, 51) -Weakness in current information system (13, 16,22, 29–31,53,60) -Lack of coherent strategy and adequate investment in electronic health (22) -Lack of human resources planning (8,17,22,23,26, 30,31, 43,60,82) -Lack of strategic management in healthcare organizations (8, 30,71) -Instability of managerial life in system (10, 30) -Lack of merit-based selection system in management (10, 30) -Centralization and Lack of delegating authority to the local levels (10, 17, 18, 36, 39, 54) -The weakness in terms of continuity and comprehensiveness of care (18, 22, 45, 63, 82, 83)	-Wide network of specialized services and outpatient services through hospitals (22) -The presence of the strong private sector in the health care system (54)	-Separated componentsof health governance (22, 59) -Providing health services by unrelated organizations (22) -Lack of defined organizational structure for disaster management, elderly and health donors (22, 29, 30) -Migration to urbanareas (12, 21, 34, 55) -Rapid epidemiological transition and changes in lifestyle and increase in accidents rate (11, 41,55, 56)
**Regulation**	**Strengths**	**Weaknesses**	**opportunities**	**Threats**
	-Appropriate regulation and programs in health and disease prevention (57, 58) -Existence of evidence-based clinical practice guidelines in family physician program (41)	-Inconsistent of some current health laws (8,24) -Lack of document regulations in some parts (8) -No respecting to existing regulations (8) -Lack of periodically monitoring of the implementation of policies (22) -Lack of effective control over providing health services (8,22,53,61) -Lack of proper legislation and super vision about private sector (8,29, 30, 43, 45, 59) -Lack of legislation about Electronic Health (22) -Weakness of pharmaceutical policies (22) -Lack of updated and defined standards about medical equipment (22) -Lack of executive and legal requirements about family physicians program (31)	-Accentuate to health andits determinants in upstream laws (60)	-Conflict of interest between policy-makers and managers (22) -Low awareness of national managers and policy makers in some health projects (61)
**Behavior**	**Strengths**	**Weaknesses**	**opportunities**	**Threats**
	-Close and intimate social relationships between Behvarz and local people (19, 20, 35,67) -Promoting healthy attitudes and behavior in local communities (35) -Satisfaction of communities about Behvarz performance (62, 63)	-Lack of the sense of ownership among community members towards health system (19) -Low community involvement in solving health problems (31, 63) -Lack of flexibility and accountability in the health system (30, 84) -Health workers insufficient training in communication skills and counseling (85)	-High literacy rates of adults (64) -Increased number of university students, especially woman (11, 21) -importance of health in the Islam (21)	-long-term drug abuse habit in Iran (11, 66,67) -Lack of comprehensive sexual relationship education for adults (68,69,70) -High rate of traffic accidents (81,86) -Incorrect lifestyle (65)
**Financing**	**Strengths**	**Weaknesses**	**Threats**	
	Free of charge services (6, 18–21)* Free medical insurance in some areas (60)	Parallel systems of financing in the system (8) Lack of coordination in financing (54) Lack of separation in financing and service delivery (54) Inadequacy and lack of financial resources (22–24) Lack of continuity in financial resources (22) Unfair distribution of resources (8, 22) Lack of coordination between the funding sources and required services package (22) lack of complete insurance system to cover the entire community (8, 43, 54)	Economical problems (8, 11, 22, 26, 27) The negative effects of the war with Iraq (8, 22,26, 27) Dependency of state budget on oil revenues (8,22, 26, 27) Inadequate investment by the private sector (22) Spending more money on the second and thirdlevels of health care services (23) Growth of health care costs (72) Emerging new diseases in Iran (72) a large refugee population (11) Aging phenomenon (11, 24, 73, 74) Non-uniform distribution of the rural population in remote areas (43)	
**Payment**	**Strengths**	**Weaknesses**	**Threats**	
	-	lack of motivation in used payment systems (29–31) Inadequate capitation fee and inappropriate allocation of it (31) The dominance of fee for services and salary payment (8, 22, 54) Inequities in payments to providers (in different levels and same levels) (8,22) Having part-time jobs, or more than one job by health workers (60, 63)	increased inflation rate (22)	

* The number in parentheses indicates the content references listed at the end of the article.

## Discussion

Financing means the way of mobilizing money and method of using it. This control knob affects some health system’s outcomes such as; health status and risk protection (12). The greatest strength of the Iranian PHC system in the financing is related to free of charge services that lead to economic access of communities (18–21). In PHC statement, was emphasized that “the cost of PHC should be payable by the community and government” (21). This prerequisite can be seen in the PHC system of Iran. Nevertheless, inadequate financial resources, discontinuity and unfair distribution of financial resources are the main weaknesses of the Iranian PHC system (22–24). However, the PHC, especially during the implementation phase (particularly in developing countries) requires great financial resources (21).

Financial problems in the health systems are the most common problem in the developing countries. Sabri (2008) in his article by the title of “Thirty years of PHC in the eastern Mediterranean region” reported that most of the countries in eastern Mediterranean region are facing limited funding (17).

Falling Gross Domestic Product (GDP) and health budget have also impacted negatively on PHC performance in many countries in Africa as well (25). Threats observed in the financing control knob are economic problems such as inflation, unemployment, the negative impact of the Iraq war (from Sep 1980 to Aug 1988), a large refugee population mainly from Afghanistan and oil dependent state budget. All of these threats could be influenced financial status of PHC system (8, 11, 22, 26, 27).

PHC system of Iran is facing the problem of shortage of funds for its programs, some issues such as; inefficient health systems and high administrative costs had been highlighted this problem (28). A variety of health service financing systems was expressed as important challenge for PHC system of Iran (29).

Payment implies the methods of paying out of money raised by financing to provider or consumer individuals and organizations (12). Lack of motivations in payment systems used in the Iranian PHC system and the dominance of salary and fee for services payments are weaknesses of payment in the Iranian PHC system (29–31). The Alma-Ata declaration statement noted that “Individual payment on a fee-for-service basis is certainly not a solution widely applied” because in the long-term will increase health costs (21). There is insufficient evidence to support the role of financial incentives to improve the quality of PHC, so financial motivations should be used with caution (32).The main threat in this area related to negative effect of inflation rate at real value of a health staff salaries and wages that make job dissatisfaction among health workers (22).

In general, various studies show that provider’s payment system was not desirable in Iran’s PHC; therefore the salary system could not be an incentive to improve performance, quality and efficiency (29).

In terms of “organizing”, the Iranian PHC system has many strong points including wide network of health centers and the formation of urban and rural branches in this network, which improves health indicators in rural areas and eliminate urban and rural discrimination (11, 21, 25, 33–38). The introduction of a new form of multi-purpose health workers called Behvarz is another achievement of the country’s health system, they are selected from the rural environment and educated in Behvarzi training center for two years and they get hired in the health-house of same village (39, 40). In PHC statement, stated that; “For many developing countries, the most reasonable solution for coverage community with essential health care is to employ community health workers who can be trained in a short time to operate specific tasks” (21) and Iran has particularly been successful in this case. Development of family physician program in rural and in the cities fewer than twenty thousand inhabitants is strength of Iranian PHC system in this area (23, 29, 31, 41–43).

Malekafzali (2014) in his sudy indicated that, one of the dramatic successes of Iran’s PHC system was comprehensive network of health care throughout the country. He emphasized that political commitment of Iranian has been led to the formation of the health network, despite the war conditions in the country (44).

On the other hand, Iran’s PHC system is also facing fundamental weaknesses in “organizing” such as mechanical organizational structure at local level that makes health system gradually weak to respond to the emerging needs of population. However, because of the multiplicity of programs and integrated instructions, PHC system is very formalized and centralized, which have reduced creativity and motivation of human resources (8, 17, 18, 30, 32, 36, 45–50). The PHC faces difficulties in urban areas such as fragmented and scattered PHC system and the lack of referral and goalkeeper system which increase parallel activities and causes ambiguity in patients (12, 17, 25, 29, 32, 33, 43, 47, 51, 52).

Another weakness of organizing is related to PHC traditional information system (paper-based) in collecting and sending data needed for policy-makers. In addition, there is a lack of adequate data management and analysis skills in health care employees (13, 16, 29–31, 53). According to PHC statement,” in order to plan and manage PHC, the right information is essential, but the collection of information has to be kept to the minimum required” (21). Besides, there is no coherent strategy about electronic health record system in Iran’s PHC system (22). The same weakness can also be seen in neighbor courtiers where primary health care system is challenged by under-utilization of the potential of electronic health strategies (16).

Moghadam (2012) in his study pointed out that centralization in decision making, poor analyzability and stratification of information system; are Iran’s PHC system challenges in organizing area (29).

Another fundamental weakness of organizing related to inappropriate human resources management and imbalance between demand and supply of health workers. For example, in some fields such as midwifery, can be seen surplus and unemployment of human resources; and in other fields such as family physician, there is a shortage of man power (8, 22, 43).

The presence of the private sector in the health care system is a big opportunity for Iranian PHC system in the area of organizing (54). In addition, the main threats in this control knob include separated components of the Iranian health governance and providing health services by some organizations that their main mission is not health service delivery such as; municipalities, banks, oil ministry, judicial system, which leads inefficiency in whole system (22). Another threat in this area is related to rapid epidemiological transition, changes in lifestyle and increasing chronic diseases prevalence, which are very important threats and requires appropriate modification in PHC structure (41, 55, 56).

“Regulation” is the use of the government power to change the behavior of individuals and organizations in the health system (10). Regulation is essential for proper implementation of PHC. In this regard, in some countries, new laws are needed or the old laws should be amended to facilitate the development of health services (21).

The main strengths of the Iranian PHC system in regulation and supervision control knobs are appropriate regulation and programs in health and disease prevention such as; the cesarean section rate reduction law in state and referral hospitals and existence of evidence-based clinical practice guidelines in family physician program (57, 58).

However, some weaknesses in this domain can be seen such as; inconsistency among some current health laws with each other and with the general policies at the country level, lack of effective control over providing health services (22), input-based assessments, lack of appropriate data (8) and lack of proper legislation about private sector behavior (29, 30, 43, 47, 59). The main opportunity of this control knob refers to existence of some articles in the Iranian constitution and the country’s twenty-year vision about the health of population and social determinants of health (60). However, the conflict of interest between policy-makers and managers (22) and low awareness of national authorities on health projects such as the family physician program (61) are important threats in this area.

Selecting Behvarz from local community, who is familiar with norms and culture of society and have friendly relations with the local people is the most important strength of Iran’s PHC system in behavior’s control knob (19, 20, 28, 35, 44, 62).

The Iranian health system has some weaknesses in this control knob, such as; lack of health system ownership sense in society members (19) and declined community involvement in solving health problems (31, 63). According to PHC statement; “community participation is the process by which individuals and families adopt responsibility for their own health and develop the capacity to participate for themselves and the community’s development” (21). Efforts have also been made to promote community participation and empowerment of society (17).

Raising adult literacy and its effect on understanding of health messages and self-care of individuals, strong support by the religious authorities based on the importance given to health in the Islamic religion are opportunities of this control knob (21, 64). There are many threats in this area, including changing lifestyles and rising obesity and chronic diseases (11, 18, 26, 65), long-term drug abuse habit among people (about one and a half million of population are addicted) (11, 66, 67), lack of comprehensive sex education for adults and learning sexual information from unreliable sources (68–70) which will have a devastating impact on community health.

## Conclusion

The Iranian PHC system has numerous successes in dealing with health system’s control knobs; which largely part of that relates to health network deployment, the role of Behvarz, health indicator improvement in rural areas and the elimination of urban-rural inequality. However, there are some weaknesses in this system, such as; insufficient financial resources and lack of the continuity of financing. PHC system in urban areas is not coordinated and the steady decline in urban health center’s users can be seen. Moreover, by considering socio-economic changes the current structure of PHC system needs to be reformed to coordinate with phenomenon of chronic diseases, accidents and aging. The current information system in PHC does not provide the required information for decision makers and policy makers, so it needs to be transformed to the electronic system with unique electronic health file for people.

## Ethical considerations

Ethical issues (Including plagiarism, informed consent, misconduct, data fabrication and/or falsification, double publication and/or submission, redundancy, etc.) have been completely observed by the authors.
